# BRAQUE: Bayesian Reduction for Amplified Quantization in UMAP Embedding

**DOI:** 10.3390/e25020354

**Published:** 2023-02-14

**Authors:** Lorenzo Dall’Olio, Maddalena Bolognesi, Simone Borghesi, Giorgio Cattoretti, Gastone Castellani

**Affiliations:** 1 Department of Physics and Astronomy, University of Bologna, 40127 Bologna, Italy; 2 Department of Medicine and Surgery, University of Milano Bicocca, 20900 Monza, Italy; 3 Department of Mathematics and Applications, University of Milano Bicocca, 20126 Milan, Italy; 4 Department of Experimental, Diagnostic and Specialty Medicine, University of Bologna, 40127 Bologna, Italy

**Keywords:** multiplex immunostaining, Gaussian mixture, lognormal, single-cell, Bayesian, dimensionality reduction, cell type, clustering, lymphoid tissue, effect size

## Abstract

Single-cell biology has revolutionized the way we understand biological processes. In this paper, we provide a more tailored approach to clustering and analyzing spatial single-cell data coming from immunofluorescence imaging techniques. We propose Bayesian Reduction for Amplified Quantization in UMAP Embedding (BRAQUE) as an integrative novel approach, from data preprocessing to phenotype classification. BRAQUE starts with an innovative preprocessing, named Lognormal Shrinkage, which is able to enhance input fragmentation by fitting a lognormal mixture model and shrink each component towards its median, in order to help further the clustering step in finding more separated and clear clusters. Then, BRAQUE’s pipeline consists of a dimensionality reduction step performed using UMAP, and a clustering performed using HDBSCAN on UMAP embedding. In the end, clusters are assigned to a cell type by experts, using effects size measures to rank markers and identify characterizing markers (Tier 1), and possibly characterize markers (Tier 2). The number of total cell types in one lymph node detectable with these technologies is unknown and difficult to predict or estimate. Therefore, with BRAQUE, we achieved a higher granularity than other similar algorithms such as PhenoGraph, following the idea that merging similar clusters is easier than splitting unclear ones into clear subclusters.

## 1. Introduction

Single-cell biology (whenever we speak of “single-cell” acquired from imaging techniques, it would be appropriate to remind ourselves that, in reality, we are talking about computational approximations of cell-segmentation boundaries and spatial-mappings of features to DAPI stained nuclei) has revolutionized the way we understand biological processes [1]. Prior to 2013, very few biological assays were indicative of single-cells (e.g., FlowCytometry); the introduction of single-cell RNA sequencing changed the paradigm. In a few words, it can be explained as the detection of the same traits in every single-cell in a sample, not just as the mean value of the bulk. Starting from the year 2000, a large group of technologies—“single-cell multi-omic technologies”—were rapidly developed. Each of them provides a very specific kind of information for every cell analyzed based on genomics, transcriptomics, proteomics, and metabolomics signals, not to mention spatial approaches that associate with the cell identity its x and y localization within the tissue. This latter, together with information related to neighboring cells [2], is crucial information because cell identity and role are determined by the spatial context. Integration of them into “single-cell multimodalomics” [3,4,5] is the next step to fully characterize cells and cell types.

While these technologies were flourishing, many global research efforts have been made to collect and share an enormous amount of single-cell data and to make them publicly available. Some large consortia have been created and now represent the standard and the repository to which we refer. Among them, the most important are the Human Cell Atlas (HCA) [6], BRAIN Initiative Cell Census Network (BICCN) [7] and Human Biomolecular Atlas (HuBMAP) [8].

This revolution has added a new level of resolution to what we can capture and enables us to better understand the cell phenotypes, the dynamics, the trajectory of their development, and ultimately the complexity of a sample. In medicine, the single-cell approach is particularly helpful, revealing unknown mechanisms in healthy and pathological tissues, and improving healthcare [9,10].

New bioinformatics tools have become more and more indispensable in order to explore single-cell data [11]. Moreover, single-cell analysis was intrinsically born as a discipline that has to face big data sets, with thousands of entities (i.e., cells) and tens of thousands of columns. All of this had to be managed with unsupervised/supervised approaches, and required different expertise. Therefore, these kinds of data are complex to deal with.

Thanks to global initiatives and to the commercialization of these technologies, the need to approach these big data from a computing point of view, in order to standardize analysis and make them available, grew exponentially. In the last few years, more than 1000 bioinformatic tools have been developed, mainly in the two world-wide used interpreted languages, R and Python [12].

High-plex spatial proteomic represents a small but crucial niche for many reasons: it has a single-cell resolution associated with spatial localization, it evaluates the presence of protein and not RNA signal (bypassing post transcriptional modification), it analyses whole cells without losing any types of them [13]—as often happens in tissue disaggregation [14] or other single-cell technologies—and it allows retrospective study on Formalin-Fixed Paraffin-Embedded (FFPE) sample collection, which is easily integrated with other “-omics” technologies.

In this project, we consider single-cell proteomics data extracted from the lymphoid tissue, which is a very dense type of tissue (approximately 1 to $2\times 10^{6}$ cells per mm ${}^{3}$ of tissue [15]).

Among the big repositories of single-cell data, it is not very often represented. When available, it contained only a selected population of cells, such as stromal cells [16] or a single organ, such as tonsils [17]. Unfortunately, with the exception of the HuBMAP project [8], there are no other lymph nodes data sets from “imaging technologies” available and, at the moment, shared data have no more than 30∼40 Antibodies.

Moreover, in the single-cell field, the majority of the most valid and interesting developed tools are specifically created for single-cell RNA sequencing data analysis [18,19] or flow cytometric data [20,21]. One of the few methods widely applied outside the original scRNAseq data is PhenoGraph [22]. The evolution of technologies [23,24] has added modules for data integration, annotation, spatial distribution, and neighborhood analysis; however, a dedicated part to approach data sets coming from single-cell imaging technologies is still missing or preliminary [25].

In short, many methods are tuned and perform clustering for high dimensional complex data, but very few of them are suited for spatial transcriptomics and the continuous property of its data. Moreover, the price to pay for spatial information is represented by a stronger neighborhood effect on each cell signal, since in some cases, cells can be tightly packed, resulting in their surface markers being picked by neighboring cell signals as well. This implies marker distributions in which the transition between different subpopulations is smoothed out, and such subpopulations are harder to split. Lastly, the high number of markers included in most databases presented during this analysis (seven out of eight datasets have 70+ markers) adds a further level of information that can and should be analyzed together. It is for all the above mentioned reasons that we aimed to develop BRAQUE, a complete pipeline tailored for spatial Immunofluorescence data sets.

## 2. Materials and Methods

### 2.1. Datasets and Data Acquisition

The data used for the analysis consisted of eight different data sets (per number of markers and cells) of normal lymphoid tissue, which were obtained with the Multiple Iterative Labeling by Antibody Neodeposition (MILAN) technology [26] (seven samples) and CODEX (one sample). MILAN datasets consist of three tonsils and three lymph nodes cores (each of 2 mm in diameter) and one whole lymph node, all of them were sections of 5 μm. The number of cells ranged from ∼25 k to ∼65 k for the six cores, while the whole lymph node dataset had ∼730 k cells. Each of these datasets counted 70+ markers after a marker selection step performed by the experts, all of them acquired with 8-bits channels. Cores belonged to three different tissue microarrays (TMA) constructed with a Tissue Microarrayer Galileo CK4500 (Tissue Microarrayer Model TMA Galileo CK4500; Integrated Systems Engineering srl, Milan, Italy). Sections were stained by using MILAN technology [26], which consists of multiple stainings, imaging and stripping cycles in immunofluorescence. Images were acquired with a NanoZoomer S60 slide scanner (Hamamatsu, Japan) at 20× magnification. This method has been shown to preserve tissue integrity and provide high stainings reproducibility (less than 10% variation) over 30 cycles [27].

Primary antibodies were validated for in-situ use on FFPE sections [28]. Highly expressed, partially overlapping, lineage-defining markers were preferred, including nuclear transcription factors. Multiplex staining and image optimization were performed according to a published protocol (MILAN [26]). The CODEX dataset was downloaded from the repository Globus (of the HubMAP project) and counted ∼109 k cells. This dataset counted only 28 markers acquired in 16-bits channels, and therefore there was no marker selection step, in order to not enlarge the already considerable difference with MILAN datasets in terms of dimensionality.

DAPI-based image segmentation was performed by means of the algorithm “CyBorgh,” developed by S. Borghesi (available upon request to the same author). It is a Matlab code which can handle very large images (in excess of 250 megapixels) in a reasonable time (it took less than 3 h on an Intel Xeon 6130 2.1 2.6 GHz 16 core, 192 GB RAM machine to segment a 1 terapixel 8-bit gray scale image with 2.2 million cells found and to generate its dataset with 92 filters). CyBorgh operates in two steps: it initially searches for the boundaries of individual tissue cells in the DAPI image after having applied to it a series of filters which must be carefully tuned to specific image features such as noise, cell size, shape and inner structure details. Once the segmentation of the DAPI image is produced, the connected components of its complement (which are expected to correspond to tissue cells) are sorted out in a Matlab cell array, each of its objects containing the coordinates of the pixels of one component. If *n* is the number of filtered images of the same portion of tissue, a stack of *n* accurately registered images is used to read out the pixel values of each component in every image. Let *k* be the number of pixels of a component. This way, the algorithm associates to that component *n* sets of *k* integers between 0 and 255. By fixing a metric (in our case it was the mean), we can “merge” each of the *n* sets of integers in a rational number, thereby producing a vector in the *n* dimensional Euclidean space. The data points comprise all such vectors, each representing one tissue cell/component in the DAPI image in such a space. Their coordinates are the “raw” data. Summing up, CyBorgh takes *n* registered images as input, one of which is declared to be DAPI, and outputs a .csv file with rows corresponding to coordinates of data points and columns to filter the response on tissue cells.

It was at this point that the continuous nature of data came in play. When the pixel is acquired, for every marker, it has a usual 8-bit discrete value. But after the segmentation step, every identified cell was made of multiple pixels, and therefore their value needed to be averaged to extract a single value for a single-cell for each marker, giving the continuous nature to our data. Moreover, single-cell data from CODEX dataset have already been observed to behave as a continuum regarding protein expression [29], reinforcing the concept that we are not dealing anymore with discrete data as in a single-cell RNA seq.

### 2.2. Data Preprocessing and Lognormal Shrinkage

In the preprocessing, we introduced a new method to improve the capability of identifying clusters in the successive steps of the pipeline.

The core idea is to look at each marker distribution within a dataset (e.g., considering only one dataset at a time) and find possible subpopulations with a similar distribution but shifted in location (therefore having a higher or lower marker expression). This concept resembles quite well the working principle of distribution mixture models.

First, we have to highlight that one of the main differences between transcriptomic and fluorescence data resides in the distribution followed by their values. In both cases, values are non-negative, but transcriptomic data follow a discrete distribution, while the data used for this study are continuous. Therefore, many algorithms and packages built for discrete data, such as Poisson, or Negative-Binomial distribution, are not suited for continuous data.

Since mixture models are often computationally heavy, to deal with our data, we had to find a distribution exhibiting the following properties: continuous, non-negative, and fast to compute and model. Therefore, we chose to use the lognormal distribution as the basic element for our mixture model. This also allowed the use of pre-built and optimized Gaussian mixture models, still for computational reasons, instead of a slower mixture model with customizable distribution.

To perform this task, we used the Bayesian Gaussian Mixture (BGM) algorithm from the scikit learn python library. The reason behind this choice is that it allows us to use variational inference, not just to infer the mixture parameters but also to infer from the data the most suited number of components to use, where each component is a Gaussian distribution with its own independent mean and variance (if “full” *covariance_type* is specified). We selected the Dirichlet process prior due to the fact that it is a priori probability distribution characterized by an infinite, unbounded number of partitions. Even if, for computational reasons, we must work with an approximation with a finite number of components, we need to specify an upper bound. This upper bound, assuming it is higher than the “true” number of components, affects only algorithmic complexity, and not the actual number of used components, since this algorithm gives a non-zero weight only to the components that are needed [30]. This property should be properly taken into account. On one hand, the higher this number is and the more accurate the algorithm estimates are (until a certain upper limit is reached). On the other hand, the smaller the number, the faster the convergence of the algorithm.

We will further show values for this parameter but, in order to generalize, we suggest a procedure to properly tune a good trade-off between accuracy and computation time: if time is not a concern, choose the smallest number of components for which every feature (or at least 95% of features ) ends up having at least one discarded component after the BGM fit procedure. If, instead, time is a concern and the previous suggested value ends up making the algorithm too slow, we suggest using the highest value that meets your time requirements, since the lower the value, the more subpopulations will risk being merged with each other (making it harder for the next steps to split them). This recommendation is not strict, using slightly bigger values for the number of components will simply imply a waste of time but equally good results, using slightly smaller values will risk sacrificing a bit of quantization, but the core of this step is just to *guess* possible initial subpopulations; the successive pipeline is in charge of validating and tuning this guess.

In the BGM algorithm, the data points are assumed to be generated from a mixture of *K* Gaussian distributions, where *K* is the number of components. Each Gaussian distribution is characterized by its mean $\mu_{k}$ and covariance matrix $\Sigma_{k}$ (which has its most general free form if we specify the “full” value for the covariance_type parameter). The mixture weights are represented by $\pi_{k}$, where $\sum_{k=1}^{K}\pi_{k}=1$.

In the Bayesian framework, prior distributions are specified for the parameters of the Gaussian mixture model, including the means, covariances, and mixture weights. The Dirichlet process prior is a non-parametric prior defined as $\pi_{k}∼DP\left(\alpha ,G_{0}\right)$, where α is a hyperparameter called a scale parameter, and $G_{0}$ is the base distribution.

Blei and Jordan proposed an algorithm for inferring the posterior distribution of the parameters of BGM using a Dirichlet process prior, as described in detail in Section 3 of their paper [31]. This can be briefly summarized in two steps:

1. Sample from the posterior distribution of the cluster assignments given the current parameter estimates;
2. Sample from the posterior distribution of the parameters given the current cluster assignments.

The algorithm is repeated until the parameter estimates converge to their stationary distribution.

For further details about the updates of the parameters mixture, or their bounds, please refer to the sci-kit learn documentation and see Section 2.1.3.2., “Variational Gaussian Mixture Models” [30].

When the fitting algorithm converged, all points were assigned to a Gaussian distribution by the algorithm. This procedure was possible by considering for each value the probability of belonging to the i-th Gaussian, computed as:

(1) $$P\left(x\in g_{i}\right)=\frac{|x-m_{i}|/\sigma_{i}}{\sum_{i}\left|x-m_{i}\right|/\sigma_{i}},$$

where $g_{i}$ is the *i*-th Gaussian component, characterized by mean $m_{i}$ and standard deviation $\sigma_{i}$. The point *x* was assigned to the Gaussian with the highest $P(x\in g_{i})$.

Once all points were assigned to a Gaussian distribution, every point was then shrunk towards the mean of the belonging Gaussian, using

(2) $$x_{new}=m_{i}+\left(x-m_{i}\right)/\gamma ,$$

where γ is a properly tuned contraction factor. This step dismembers the original distribution, but preserves the order. Doing so, values rankings are maintained but gaps are created in correspondence of where we could have a good separation between two different subpopulations.

A small incompatibility could be that the lognormal distribution is strictly positive instead of non-negative, but this problem was easily solved by adding a very small constant before fitting the mixture model. Doing so, one of the Gaussian components from the mixture model was always going to account for the values at the very small value, basically identifying as a separate subpopulation all those values that started as a zero in the original distribution.

A summary of the preprocessing algorithm for each single marker could be as follows:

1. Robustly scale the marker distribution, dividing it by the median absolute deviation (MAD). This step is suggested so all the parameters for the Bayesian Gaussian mixture algorithm are going to be the same for every marker;
2. Sum a small positive constant to every value to avoid taking the logarithm of 0;
3. Compute the logarithm of the shifted and robustly scaled distribution;
4. Perform a Bayesian Gaussian mixture fit using variational inference algorithm;
5. Once the final Gaussians are identified, shrink every value towards its belonging Gaussian, then back transform the values by exponentiation;
6. Subtract the minimum of the distribution and (optionally) scale robustly, dividing again by the new MAD of the final distribution.

We named this preprocessing Lognormal Shrinkage (LNS).
Two final considerations regarding the LNS method:

- The shrinking factor was tuned based on the quality of results, but no big difference in the range from 2 to 10 was observed, therefore, a value of 5 was chosen;
- The base for the logarithm does not affect the performances, only scales the log transformed distribution; therefore, we used base 2 logarithm and, in the case of different choices, the shrinking factor should be tuned accordingly (e.g., the base 10 logarithm should use a contraction factor of 5/ln(10)∼2).

**Figure 1:**
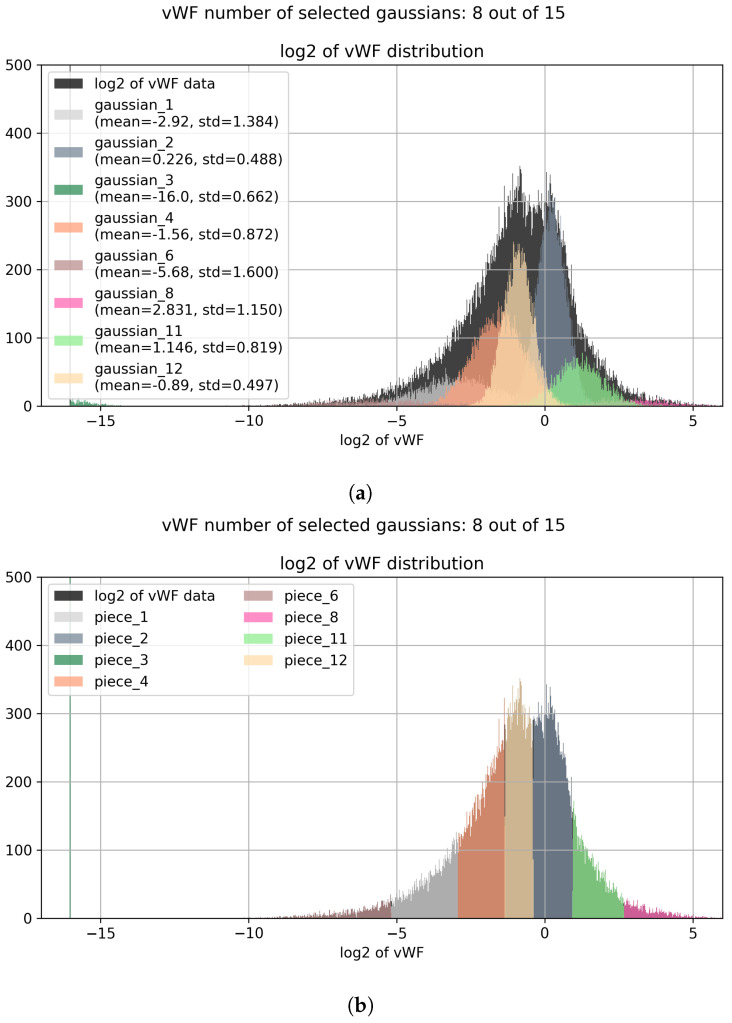

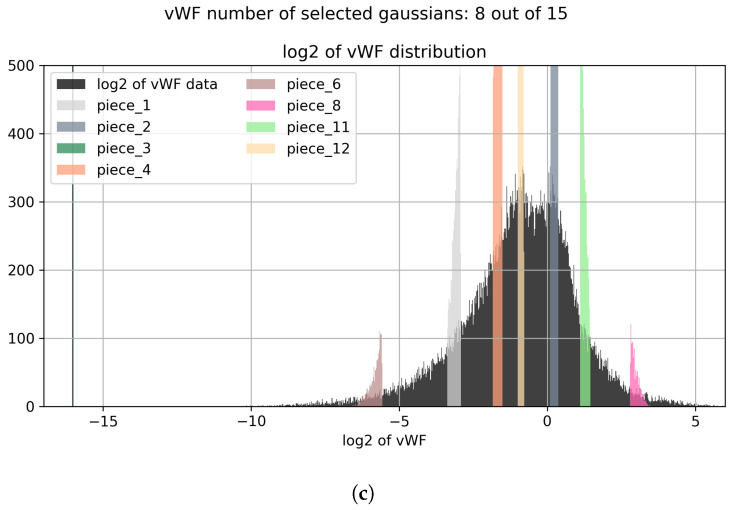
This Figure shows the Lognormal Shrinkage effect on the log2 of the vWF distribution. As we can see, the algorithm automatically selects the best number of components instead of using all of the available ones. Then a clear separation between different subpopulations is achieved, in order to help further UMAP embedding in creating a more fragmented output for an easier and more precise clusterization. (**a**) Bayesian Gaussian Mixture fit. (**b**) Assignment step. (**c**) Shrinkage step.

An example of the effect of the LNS procedure on the distribution of data is reported in Figure 1.

### 2.3. Dimensionality Reduction Step

The dimensionality reduction step has the aim of moving data from a high-dimensional space to a lower-dimensional space, called embedding, in order to tackle data sparsity and other problems caused by the course of dimensionality.

For this step, Uniform Manifold Approximation and Projection (UMAP) [32] was chosen due to its huge advantages in terms of memory and computation time with respect to t-SNE (t-distributed stochastic neighbor embedding) [33], and its capability of wrapping more information than older methods such as multidimensional scaling (MDS) [34]. Two main parameters that must be fixed before running UMAP are the number of nearest neighbors *K* and the metric used to compute the distance. For the first, higher values aim to produce a much more characterized embedding by the global structure. A value of 50 (instead of the default 15) was used to have a good trade-off between global structure and computational efficiency. Regarding the metric, after many considerations and few trials, we decided to use the default Euclidean metric.

The UMAP algorithm can be summarized into two main phases. First, a weighted directed graph is built to represent the data neighborhood relations in the starting high dimensional space. A graph is made of nodes (which represents data points) and edges which connect two nodes, where in our case this connection represents a proximity relation between the two connected nodes. The fact that the graph is weighted means that not all edges are equal; in fact, they might be more or less strong according to an intensity value called *weight*, which resembles higher or lower proximity among the connected nodes. Directed simply implies that the weight $w_{ij}$ of the link connecting node i to node j can differ from the weight $w_{ij}$ connecting j to i. In UMAP the weight $w_{ij}$ between point i and j is computed using the equation:

(3) $$w_{ij}=e^{-\max\left(0;d_{ij}-\rho_{i}\right)/\sigma_{i}},$$

where $\sigma_{i}$ is tuned such that

(4) $$\sum_{j=1}^{k}w_{ij}=log_{2}\left(k\right)\;\;\mathrm{and}\;\;\rho_{i}=\min_{j\in \{1,\ldots ,k\}}\left(d_{ij}>0\right).$$

Here, $d_{ij}$ is the distance according to the metric in the high-dimensional space, $\sigma_{i}$ is called the scaling factor for point i and it is used to normalize the sum of the outgoing weighted edges, and finally $\rho_{i}$ is the smallest strictly positive distance between point i and its neighbors j∈{1,…,k} (and therefore $\rho_{i}$ > 0).

The second phase of the algorithm is the low-dimensional space (called embedding) optimization. This is needed to reproduce as correctly as possible the weighted directed graph structure. This procedure is achieved by placing the data points in the embedding space (either with random starting positions or with coordinates initialized by some criteria, for example via spectral embedding or, if not possible, with PCA coordinates), and then alternatively apply to every point i attractive forces towards its nearest neighbors and repulsive forces towards its non-nearest neighbors. The intensity of both attractive and repulsive forces can be tuned among the initial set of UMAP parameters. The final output of the algorithm is a low-dimensional space which replicates the proximity observed in the original high-dimensional space among the data points, but with a considerable boost for clustering algorithms performances and with the advantage of possibly visualizing the new data space.

### 2.4. Clustering Step

All clustering algorithms have advantages and disadvantages, but we will try to briefly explain the reason for our suggested choice. Hierarchical clustering brings the obvious advantage of producing a hierarchical structure that can be investigated, allows the plotting of dendrograms, and follows the very simple intuition of merging the two closest elements together iteratively.

One of its possible drawbacks is related to the linkage method, which affects the shapes of the resulting clusters [35]. In fact, it is well known that single linkage produces, for example, narrow line-like clusters, while complete linkage produces more spherical clusters. In general, even if more and more linkage methods can be found, all of them imply some kind of assumption regarding the resulting cluster’s shape. One possible solution to this drawback is switching to a density-based clustering algorithm. The most renown of this kind is Density-Based Spatial Clustering of Applications with Noise (DBSCAN) [36], which requires two input parameters: ϵ and min_samples. The first defines what is close, the second defines more or less the number of points needed to start the formation of a cluster.

Thanks to the usage of a distance parameter and a certain number of points that must be contained in this distance, DBSCAN uses density to form its clusters, allowing any possible shape with no restrictions. The big downside in this case becomes the fact that now the threshold is imposed on the density, and therefore identifying clusters of different densities could become a problem, where the bigger the difference between densities and the fewer the chances to identify both clusters at the same run.

To briefly explain DBSCAN for further purposes, its procedure could be summarized in the following way:

- Given the two parameters, epsilon and min_samples, a point *x* is defined as a “core point” if and only if at least min_samples points are within ϵ distance from *x*;
- Connect all “ϵ-reachable” points. Where two points are said to be “ϵ-reachable” if they are in each other’s neighborhood, and points are “density-connected” if they are directly or transitively “ϵ-reachable”;
- We conclude labeling as a cluster every maximal “density-connected” subset of the data, while the remaining unlabeled points are relabeled as noise at the end of the algorithm.

The HDBSCAN algorithm [37] is the hierarchical improvement of DBSCAN, and basically provides advantages from both approaches, while limiting their drawbacks. It allows the detection of clusters with generic shapes and possibly very different densities, as long as such densities are higher than the respective neighborhood. Therefore, this last algorithm is the suggested algorithm for the clustering phase. HDBSCAN’s working principle is similar to running DBSCAN for all ϵ∈[0,+inf[, then uses the different values of ϵ (or more precisely, a new distance, called Mutual Reachability Distance and based on the centroid of the neighborhood) to connect points in a hierarchical dendrogram fashion. Finally, clusters are extracted, not by cutting the dendrogram at a given depth, but instead considering cluster stability at varying depth. This last step is performed using λ=ϵ−1, and then identifying for each candidate clusters their birth (the λ at which cluster forms) and their death (the λ at which the cluster splits into separate clusters).

Now, for each point in the cluster we can define $\lambda_{p}$ as the value at which that given point leaves the cluster. Therefore, $\lambda_{p}\in ]\lambda_{birth},\lambda_{death}]$, since each point either leaves the clusters before death or exactly when the cluster splits into minor clusters. Now, we can calculate the stability of cluster C as follows:

(5) $$\sum_{\forall p\in C}\lambda_{p}-\lambda_{death}.$$

The cluster extraction is then performed by starting from leaves clusters, and replacing some of them with their roots cluster every time that root cluster stability is bigger than the sum of the leaves cluster stability.

Last but not least, one could choose to select much simpler methods such as DBSCAN for computational reasons, but it turns out that, for big databases, the performances of HDBSCAN are better in terms of computation time [38], and therefore HDBSCAN is also preferable under this point of view.

### 2.5. Cluster Characterization Step

After the clusters are identified, a method to describe which markers are mostly expressed from the clusters is needed. A first step was to perform a Welch t-test for each marker, using the null hypothesis of the cluster mean being not significantly greater than the mean of remaining cells in the sample. Here, we would like to underline that we considered a one-tailed *p*-value, since we are not interested in significantly least expressed markers, but only in the significantly most expressed ones.

An important digression should be made regarding the *p*-values at this point. It is quite common to find in the literature the usage of a standard threshold of 0.05, but we have to notice how the *p*-value is always a function of the number of samples used in the test. In particular, the more samples, the smaller the *p*-value (if a difference between the groups is present). Therefore, samples with a considerably high number of cells (e.g., $10^{5}∼10^{6}$) would end up emphasizing even very small differences, often producing *p*-values < $10^{-100}$.

It seems indispensable to understand if a marker is significantly more expressed in a cluster with respect to the whole sample, but it is also important to properly quantify the magnitude of such difference. For this reason, together with a Welch’s t-test with a Bonferroni multiple test correction, a measure of effect size was used to quantify how much a marker is more expressed in the considered cluster. Thanks to this measure, it was possible to rank for every cluster the markers from most expressed to least expressed.

A further advantage of this approach was the capability of looking for gaps in the ranked effect sizes, and defining markers probably expressed by the cluster, called tier 1 markers, and clusters possibly expressed by the cluster, called tier 2. The tiers were identified by first looking for the two biggest gaps in the positive ranked effect sizes, and then calling tier 1 the markers whose effect size was higher than the first gap, and tier 2 the markers whose effect size was between the two identified gaps.

To calculate the effect size, a robust version of the Cohen’s d was used [39], which is computed as:

(6) $$d:=\sqrt{\frac{{\left(m_{2}-m_{1}\right)}^{2}}{\sigma_{1}^{2}\cdot \frac{N_{1}\;+\;N_{2}}{N_{1}}+\sigma_{2}^{2}\cdot \frac{N_{1}\;+\;N_{2}}{N_{2}}}},$$

where $m_{i}$, $\sigma_{i}$ and $N_{i}$ indicate respectively the mean, the variance, and the number of samples from group *i*, having usually that group 2 was the analyzed cluster, and group 1 was the whole sample excluding the analyzed cluster. One last detail to notice is that the formula is symmetric with respect to the two groups, therefore from this version of *d* it was not possible to understand if a marker was more or less expressed than the whole sample; for this reason, the final metric used the formula:

(7) $$d_{signed}=sign\left(m_{2}-m_{1}\right)\cdot \sqrt{\frac{{\left(m_{2}-m_{1}\right)}^{2}}{\sigma_{1}^{2}\cdot \frac{N_{1}\;+\;N_{2}}{N_{1}}+\sigma_{2}^{2}\cdot \frac{N_{1}\;+\;N_{2}}{N_{2}}}},$$

allowing negative values of $d_{signed}$ when $m_{2}<m_{1}$.

### 2.6. Comparison with Other Existing Algorithms and Final Validation

As last step, we compared the outcomes obtained by BRAQUE with the ones obtained using PhenoGraph [22] R package, which was run on RStudio (R version 4.0.2).

The cell type assignment for each BRAQUE cluster was based on a list of its most significant markers in terms of ranked effect size. For PhenoGraph, a heatmap of marker expression by cluster was used to assign a cell type, as performed in previous work using the same algorithm [27]. Then the evaluation considered the number of noisy/unclear clusters, rare populations correctly clustered as separate clusters, and the number of redundant clusters (where this was considered as a minor side effect as far as the clusters were explainable as a correct cell type).

The corresponding Python3 code for BRAQUE will be available at https://github.com/LorenzoDallOlio/BRAQUE from 1 March 2023.

## 3. Results

In this section, we will show the results that can be achieved using BRAQUE’s pipeline. For clarity purposes, we will not show every produced plot (since in total we produced at least three plots for each of the 620 clusters), but we will focus on explaining the key steps and showing the results for: one preprocessed marker, one whole sample analysis, one cell type across different samples, and a comparison with the PhenoGraph algorithm.

For the analysis, a server with Intel(R) Xeon(R) CPU E5-2620 v4 2.10 GHz was used, with 32 cores, 252 GB of RAM and Python version 3.9.12. In terms of memory consumption, the average memory required for the analysis was around 30 GB for the biggest database (∼730 k rows × 80 columns). Moreover, the usage of UMAP’s parameter “low_memory” set equal to “true” allowed it to never exceed 100 GB of RAM usage, required only for the nearest neighbor search phase of the algorithm (which took approximately 1 h). In terms of time required to run the analysis on the biggest database, approximately 95% of the computation time of the pipeline resides in the Bayesian Gaussian Mixture fitting procedure, which took around 2 h per column, resulting in a week of computation time for the whole analysis. It is important to underline that both memory and time consumption are not linear with respect to the number of rows in the database; therefore, the smallest databases (the cores with a number of rows between 20 k and 70 k) were computed with around 10 GB of memory, a peak of 30 GB, and with the whole analysis completed in approximately 3∼8 h.

Fine tuning of the parameters was obtained by a continuous and mutual feedback between bioinformatics and pathologists regarding computational needs, results clarity and interpretability. As in every machine learning tool, parameters should be tuned based on the nature of the data, the desired result quality, and computational efficiency. In Table 1, we briefly report suggested ranges and values that were tuned during this analysis.

Some small clarifications about the values reported in Table 1:

- Regarding BGM *covariance_type*, UMAP’s *init*, and HDBSCAN *cluster_selection_method* should be fixed to the suggested value, since any alternative could only imply worse results in terms of correctness and generalization;
- Regarding BGM *n_components* and *tol*, the best values would be as high as possible for the first and as low as possible for the second, but what we observed is that such exaggeration has strong computational drawbacks, and therefore a suggestion is to use a relatively low *tol* and tune the number of Gaussians following the criteria described in the methods section;
- All the other parameters should be tuned dataset-wise for better performances, but we can point out that, as long as values are in the suggested range, *contraction_factor*, UMAP’s *metric*, *min_dist* and *nn* should not strongly affect the results;
- Regarding HDBSCAN *min_samples*, we suggest it could be tuned as a proportion of the considered dataset. At the same time, to avoid finding excessively small clusters, we suggest to put a lower limit on such parameter, such as 10, to avoid micro-clusterization that could lead to huge number of clusters (e.g., ≥10,000);
- Unreported parameters were used with their default value.

Lastly, if time is again not a concern, we strongly recommend fixing BGM’s *max_iter* as high as possible to always reach convergence criteria, otherwise compute for the maximum amount of time possible and set this parameter consequently.

Given the structural similarities that few very spatially near populations presented, we found more correct results separating the cluster into two phases. The first phase uses `excess of mass’ (’*eom*’) parameter to find more connected components in the UMAP embedding. The second phase was a clustering repeated on the biggest cluster, and used `leaf’ as the cluster selection method and the suggested *min_samples* value times 10. This second step improved separation between some CD4+ T-cells and some B-cells populations, which consistently ended up in the same cluster for BRAQUE and PhenoGraph. This expedient could be significant to the whole pipeline or just needed to compensate the extremely tight spatial distribution that these cells had in considered data sets. Since this was the case also for the CODEX dataset, we decided to mention this approach, and the publicly available code will have a specific parameter to turn on or off this particular behavior, since we still do not know if the two populations are generating a strong cross-contamination in other tissues or only/mostly in Lymphoid tissues.

### 3.1. Lognormal Shrinkage on a Marker

Now, to show the way BRAQUE preprocessing works, we will show step-by-step the effect of Lognormal Shrinkage on the marker *vWF*, a marker used to identify “Endothelium” kind of cells, a rare, sparse but well-defined cell type.

**Figure 2:**
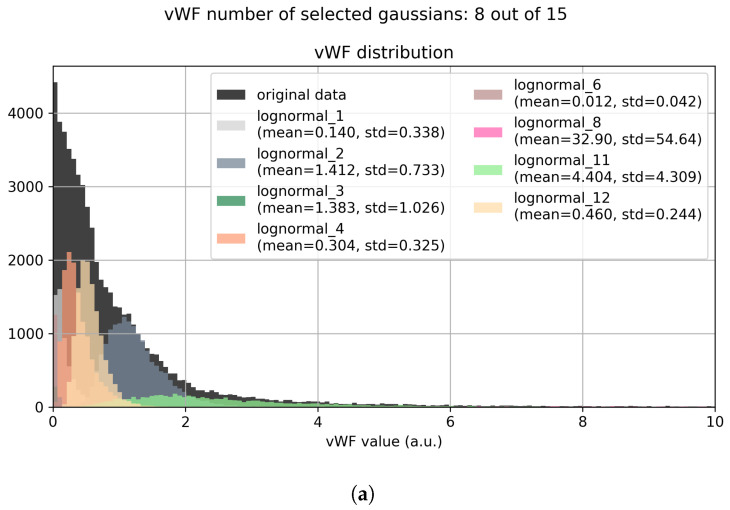

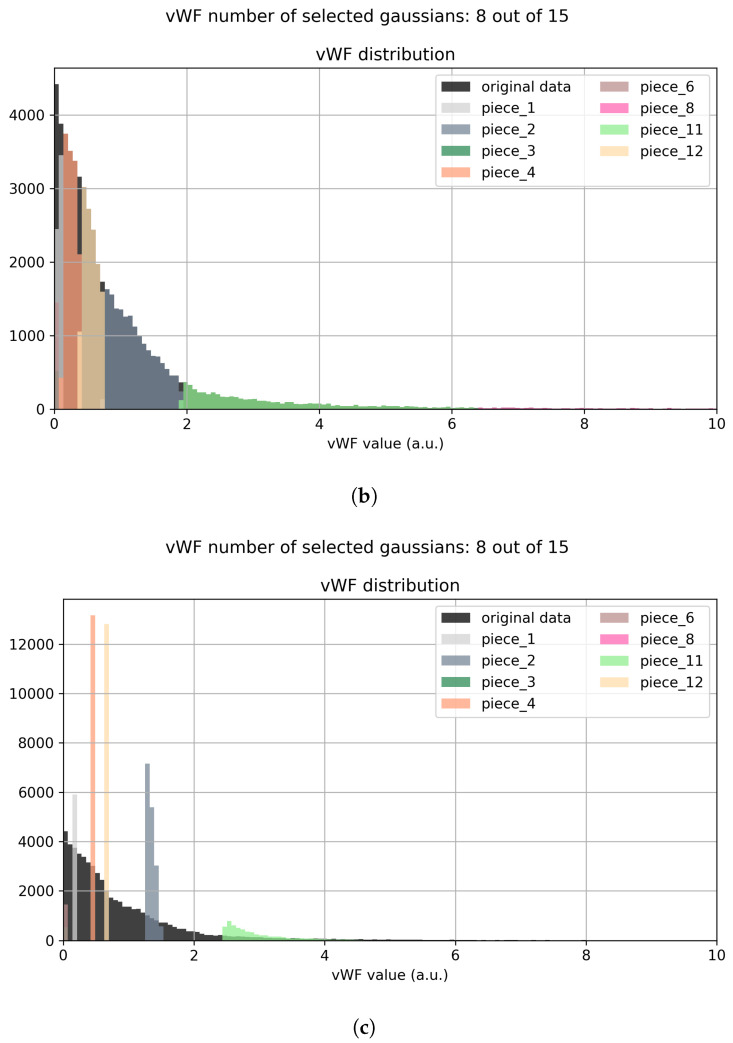
This Figure shows the Lognormal Shrinkage effect on the original distribution of the vWF marker in the dataset L2. As we can see, the algorithm automatically selects the best number of components instead of using all of the available ones. Then a clear separation between different subpopulation is achieved, in order to help further UMAP embedding in creating a more fragmented output for an easier and more precise clusterization. (**a**) Bayesian Gaussian Mixture fit. (**b**) Assignment step. (**c**) Shrinkage step.

As previously mentioned in the methods section, the procedure is performed on every marker separately. For each marker, a robust scaling is performed, dividing by the MAD. This to have all the markers in similar ranges, and therefore having the fitting parameters behave similarly among them. Then, a small constant is added (to avoid further logarithms of 0) the logarithm is taken for every value, the Bayesian Gaussian mixture algorithm is fitted using variational inference algorithm (Figure 1a). When the fit converges, each cell is assigned to the component to which it is most likely it belongs (Figure 1b), and its marker value is modified by shrinking it towards the mean of the assigned Gaussian distribution (Figure 1c). The same effect can be observed on the backtransformed marker distribution (Figure 2), reminding us that every normal distribution in the log2 space becomes a lognormal distribution in the backtransformed space, with new mean and standard deviation given by

(8) $$\begin{array}{cc} \mu_{lognormal} & =e^{\left(\mu_{normal}+\frac{1}{2}\sigma_{normal}^{2}\right)} \end{array}$$

(9) $$\begin{array}{cc} \sigma_{lognormal} & =\sqrt{\left(e^{\sigma_{normal}^{2}}-1\right)\cdot e^{\left(2\mu_{normal}+\sigma_{normal}^{2}\right)}}, \end{array}$$

where the pedix normal indicates the normal distribution in the log2 space, while the pedix lognormal indicates the lognormal distribution in the original data space.

### 3.2. Analysis of a Sample

To provide a broad view of the analysis, we will now show the step-by-step pipeline application to a whole sample, in this case specifically to the dataset L2.

**Figure 3:**
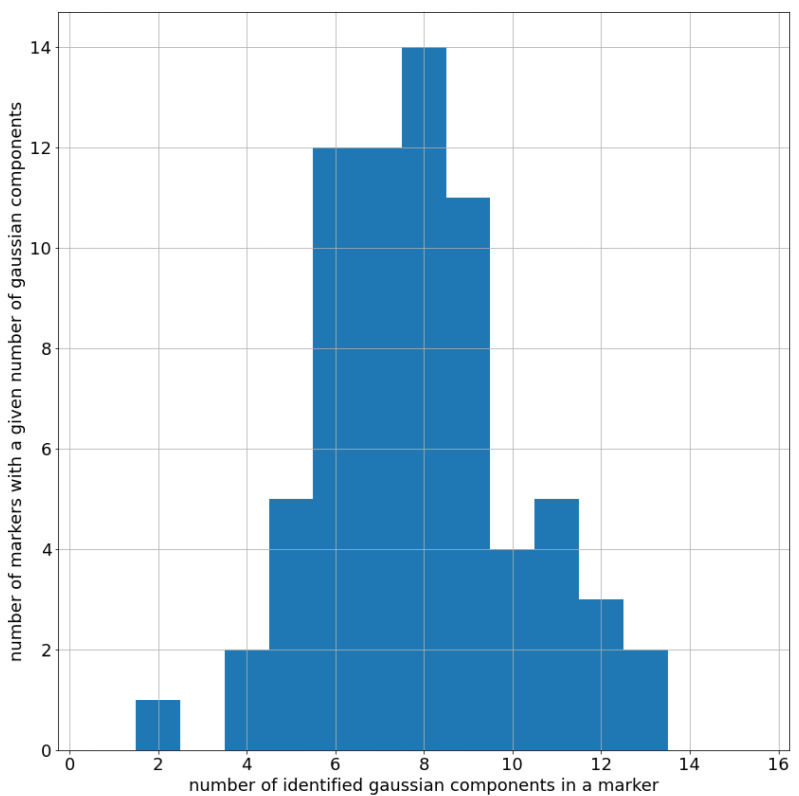
Distribution of the number of components selected for a single marker after the Bayesian Gaussian Mixture fit on dataset L2. Please notice how no marker had 15 components, showing that the upper limit of 15 given as input to the algorithm was appropriate.

First of all, in Figure 3, we show that the number of Gaussians used (i.e., 15) was sufficient, given that no marker exceeds the 14 different Gaussians with non-zero weight after the fitting procedure.

**Figure 4:**
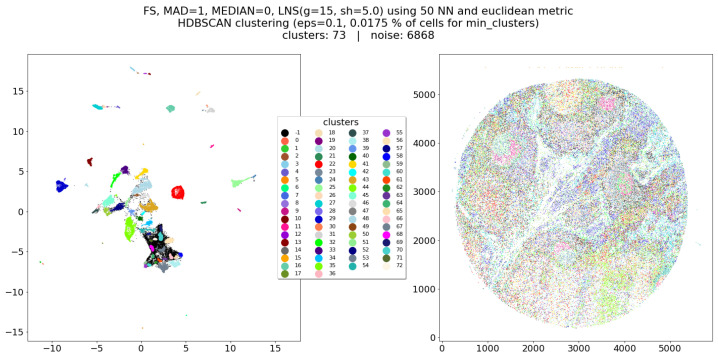
HDBSCAN clusters computed over UMAP’s embedding of dataset L2 (**left**) and reported on the real spatial coordinates of the cells (**right**).

**Figure 5:**
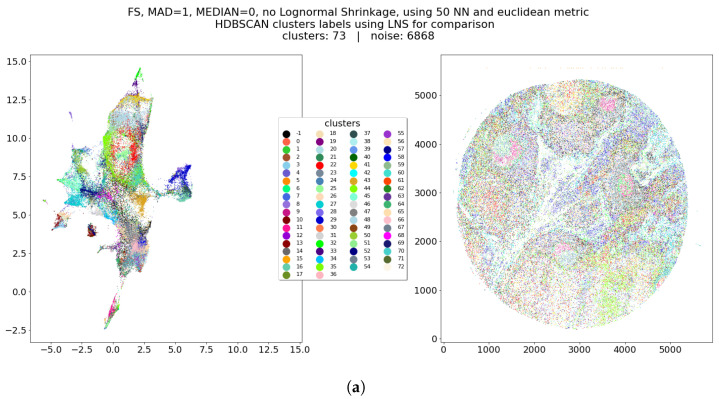

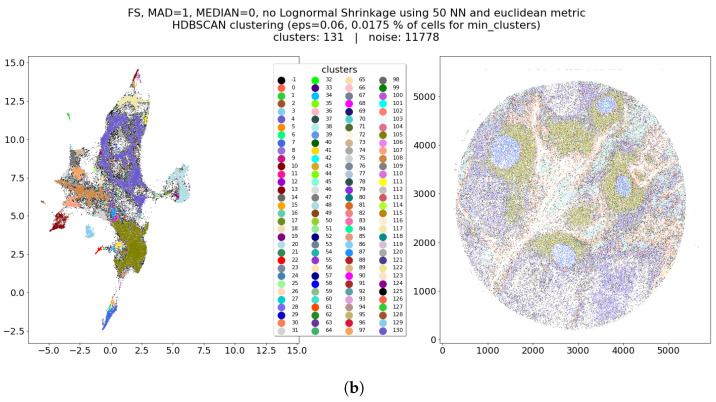
UMAP’s embedding of non LNS preprocessed dataset L2. This figure should be compared with Figure 4 to understand the higher separation that is clearly achieved at a visual level by UMAP’s embedding thanks to the insertion of BRAQUE’s preprocessing. Moreover, it is possible to compare clusters found after applying LNS (**a**) with clusters that can be find without LNS (**b**). From such comparison it seems that LNS-driven clusters are still neighboring cells in this much compact embedding, but the non LNS-driven ones result in being much more difficult to separate. (**a**) UMAP embedding of L2 with no LNS preprocessing. Clusters from the LNS preprocessed scenario for comparison. (**b**) UMAP embedding of L2 with no LNS preprocessing. Clusters from application of HDBSCAN on this embedding, please notice bigger clusters, more noise, and more clusters with respect to figure (**a**).

The main results are, after the application of the Lognormal shrinkage preprocessing to every marker, the application of UMAP on the preprocessed dataset, and the application of HDBSCAN clustering on the UMAP embedding (Figure 4). For evaluation, we ran UMAP with the same identical parameters, but on the data which did not undergo the LNS step (Figure 5). From the comparison of these two figures, it seems clear how the Lognormal shrinkage procedure added a considerably higher separation in the UMAP embedding, that is not depending on UMAP’s parameters. Moreover, it seems that LNS-driven clusters are still neighboring cells even in the embedding not affected by LNS preprocessing (meaning that those cells are preserved in respective neighborhoods), while the non LNS-driven ones result in being much more difficult to separate from each other. As can be noticed in Figure 5b, at the same time there are big clusters merging plausibly different things, many small clusters, and a higher noise (unlabeled cells), therefore any tuning of the algorithm would lead to either more noise, bigger cluster, or even more small clusters, making its proper tuning much harder and complicated to reproduce/generalize.

**Figure 6:**
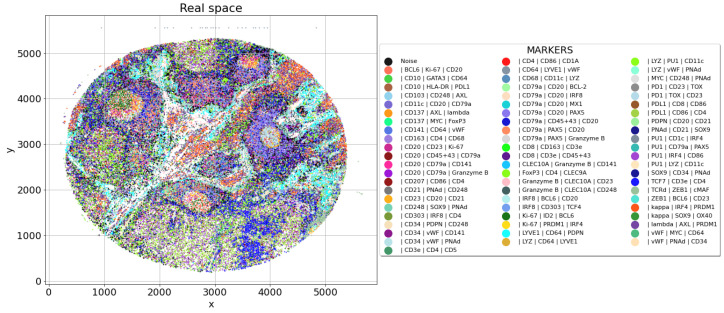
Global validation plot for dataset L2, list of most expressed markers per cluster (according to robust effect size measure). In this case, the validation is not in depth but at a global level, since the clinicians can look for known biological structures.

Once the clusters are identified, it is possible to perform the validation for both, single clusters and global results. Since the single cluster step will be the focus of the next subsection, now we will focus on the global result. This step can be performed with a scatter plot on the real space, where every cluster has an assigned explanatory label. Such a label could be the list of the main expressed markers in terms of size effect (Figure 6).

The spatial reconstruction of the clusters can help clinicians in the global evaluation. For example, according to the experts Figure 6 highlights a dense lymphoid tissue, bisected by a stromal streak and containing four lymphoid follicles, surrounded by interfollicular spaces. Moreover, it is possible to observe that three of the follicles are centered by rounded Germinal Centers (proliferating B cells).

### 3.3. Clusters Analysis

After the pipeline is performed, it is possible to analyze the single identified clusters. We will show the results for four clusters that were labeled as “Endothelium,” considering four different samples: L2, whole lymph node, T3, and CODEX, in order to show different organs and different datasets in terms of data acquisition.

**Figure 7:**
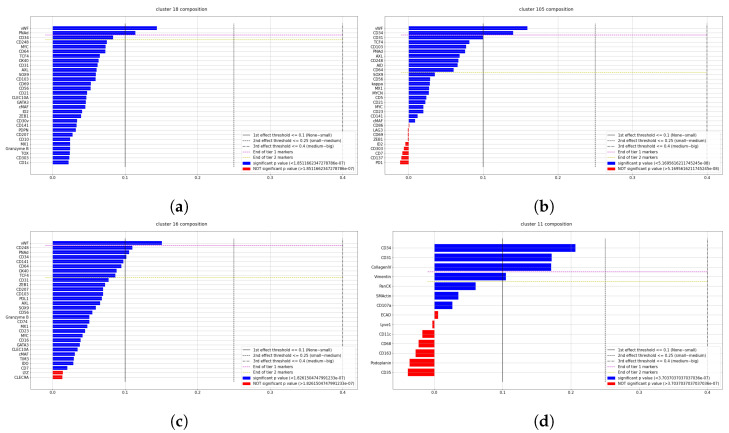
This Figure reports markers expression of 4 clusters whose cells were identified as Endothelium. The markers are ranked according to the robust effect size metric $d_{signed}$, and colored according to their Welch t-test *p*-value. In every plot it is possible to notice gaps in the descending order of markers, such gaps were used to suggest important (i.e., “Tier 1”) and possibly useful (i.e., “Tier 2”) markers for the experts following classifications. (**a**) 2 mm core of lymph node (56,962 cells). (**b**) Whole Lymph node dataset (727,729 cells). (**c**) 2 mm core of tonsil (65425 cells). (**d**) CODEX Dataset (188,450 cells).

**Figure 8:**
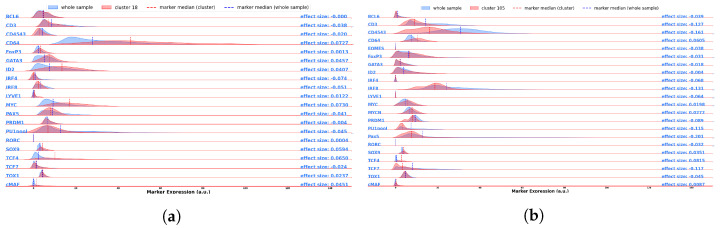

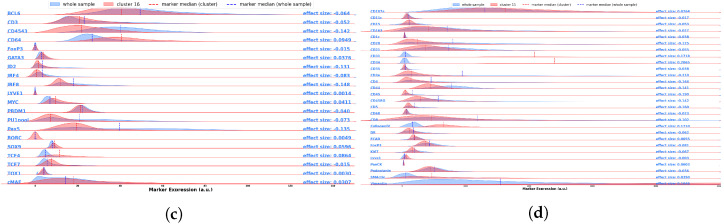
This Figure reports the comparison between whole dataset marker distribution (blue) and the cluster’s one (red). This plot is intended to help the experts in comparisons, to better show them where the actual difference comes from, and help them estimating/validating the cellular type of the cluster. The difference on the x-axis range for CODEX dataset is a consequence of that data being acquired over 16-bits channels, while MILAN data were acquired with 8-bits channels, therefore they can span from to 0 to, respectively, $2^{16}-1$ and $2^{8}-1$. Plots are resized consequently. (**a**) 2 mm core of lymph node (56,962 cells). (**b**) Whole Lymph node dataset (727,729 cells). (**c**) 2 mm core of tonsil (65,425 cells). (**d**) CODEX Dataset (188,450 cells).

**Figure 9:**
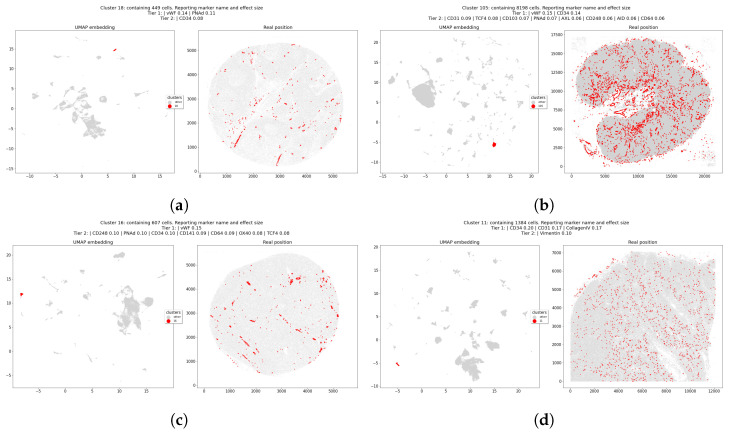
This Figure reports spatial positions in UMAP embedding (left plots) and in real space (right plots). This plots are enriched by the subtitle, which reports Tier 1 and Tier 2 markers, together with their effect size. This kind of plot was considered as the main tool for the experts to rapidly assign a cell type to the cluster, using all of the available information in one plot. (**a**) 2 mm core of lymph node (56,962 cells). (**b**) Whole Lymph node dataset (727,729 cells). (**c**) 2 mm core of tonsil (65,425 cells). (**d**) CODEX Dataset (188,450 cells).

The usual cluster report consists of three plots for each cluster, comprising the most expressed markers for each cluster (Figure 7), the expression of some selective diagnostic markers including transcription factors (Figure 8), and cluster location on UMAP plot and on the tissue (Figure 9). The last one also contained a summary of Tier 1 and Tier 2 markers, and was used by experts for a fast classification, given the fact that contained both spatial information and phenotype information. The first two were used to adjust the classification by analyzing deeply markers expression, either to better visualize the gaps between Tier 1, Tier 2, and the rest, or to inspect possible incongruities such as CD4 and CD8 both expressed by the same cluster. Cell type assignment was based on all of them and performed by experts.

### 3.4. Clustering Algorithms Comparison

After running the complete BRAQUE analysis on the eight datasets, the experts evaluated every cluster interpretability and the most suited cell type (where possible). Then the PhenoGraph algorithm was run, the clusters analyzed similarly, and the two algorithms were compared.

Among all the datasets, experts identified a total of 46 cell types, 15 of which were considered as infrequent due to their low total clusters count (≤10, among 620 clusters in total). The properties considered in the algorithms comparison were: number of different infrequent populations identified, number of different common populations identified, percentage of unclear clusters cells, percentage of T cells that are unseparated between CD4+ and CD8+ (meaning that within the same cluster we have CD4 positive T cells mixed with CD8 positive T cells). Obviously, the first two properties should be as high as possible, since identifying more cell types after expert validation is better. Moreover, these properties were considered, since validating and counting cluster-wise cell types is easier to check than verifying the correct cell type, cell-wise, for more than a million cells. On the other hand, the last two properties should be as low as possible, given that clearly separated T cells are better than merged ones, and identifying a cluster of potentially interesting cells should ideally always end up being recognized as a cell type.

On this last point, an important focus should be pointed to the noise cluster that density-based clustering methods always have, i.e., the cluster labelled as “−1”. This cluster collects all the *unclear* cells, acting as a proper bin.

The noise-cluster of HDBSCAN helps to remove cells that would average cluster properties, making them less clear. As long as this cluster’s size is relatively small (e.g., <20% of the sample cells), this property was preferred by the experts as a tradeoff for having clearer and faster interpretation. Given that all of the important cell types were found during the analysis, the noise cluster was not considered a strong negative downside, while a strong negative property was labelling a cluster as potentially interesting and then having the experts labelling it as junk. Therefore, the percentage of unclear clusters cells ignores the “−1” cluster, since it does not steal time for a useless evaluation by the experts.

Clustering comparison results are reported in Table 2.

## 4. Discussion

In single-cell analysis, many efforts have been made to standardize the process of data analysis. However, even if the majority of the tools available share the same goals, each type of technique presents its own peculiarities and involves different approaches.

In this paper, we addressed the need to have a more tailored approach to analyze single-cell data coming from imaging technologies revealed with immunofluorescence technology. We proposed BRAQUE, an integrated and novel approach spreading from data pre-processing to phenotype classification. We tested it on lymphoid tissue (tonsils and lymph nodes), which is one of the densest and most challenging tissues [15].

We introduced two innovations that we hope will become standards in the field of single-cell analysis: to “fragment” data input distribution in order to help clustering even small differences in separate clusters, and the usage of effect size measures to rank markers according to their importance in characterizing every cluster. The former could be performed in different ways. The aim of the method we chose (i.e., Lognormal Shrinkage) is not to correctly find all the subpopulations in each marker, but to guess where most relevant differences could lie and make them more evident. For instance, if two subsets are slightly different by just one marker, they will probably end up in the same cluster anyway. However, if these hypothetical subsets are slightly different on 30+ markers, then our approach helps to increase such discrepancies, making it more likely to split those subsets into separate clusters. Therefore, the key point is not finding the perfect subpopulation split, but rather finding multiple reasonable guesses, which will end up confirmed or not by UMAP embedding.

Thanks to its preprocessing, BRAQUE detects more clusters than other established methods such as PhenoGraph. Comparing between Figure 4 and highlights, feeding UMAP with our preprocessed data results in a higher number of clearly isolated data clusters.

Since the number of total cell types in one lymph node detectable with these technologies is difficult to estimate, we suggest an over-fragmentation rather than an under-fragmentation. This follows the principle that it is easier to merge similar clusters than to split unclear clusters into clear subsets. Therefore, BRAQUE may be the most suited available tool for the task of cell type clustering, with no contraindications stopping it from being extended to other types of tissues acquired with similar techniques. Therefore, we also strongly suggest privileging granularity over its opposite, in the development of a new method that will come.

Another key advantage of BRAQUE is that, in some cases, groups who dealt with analogous databases [29,40] relied on prior knowledge of markers known to have high or low expression in specific cell types, discard of non-contributory markers, neighboring cell definition, hand-gating, etc. BRAQUE instead proposes a marker-agnostic and spatial-agnostic approach with top level granularity, which we believe is more adapted to analyzing very dense and complex spatial protein data with no strong assumption or predefined bias.

An interesting further work could be applying only BRAQUE preprocessing (i.e., Lognormal Shrinkage) to the input of other clustering methods and assessing the effective gain that could come from it.

Nowadays, we too often see the usage of just *p*-values without their respective effect size. This trend should be highly reconsidered, since *p*-values are good only for Boolean/threshold-like answers regarding the statistical significance of possible differences. The problem is that, in medicine, biology, and many other fields, after the statistical significance is achieved the important question becomes “How much is it different?”, and *p*-values are not adequate for addressing such an issue [41,42,43]. For this reason, when it comes to defining which markers mostly characterize a cluster, we strongly suggest using effect size measures, whether they be of a different or similar nature to the one used in BRAQUE’s pipeline. Furthermore, the choice to classify each cluster not just by listing absolute value normalized markers, but by ranking the most significant ones, has produced a more precise classification with more cell types and more accurate phenotypes, according to the experts.

Lastly, since most of the computation time comes from the Bayesian Gaussian Mixture fitting procedure, it is important to reduce the number of maximum Gaussians as much as possible, as previously remarked in the methods section. However, in order to maintain the correctness of the approach and the quality of the results, this number should never, or almost never, “saturate” (meaning that the final number of fitted Gaussians with a weight different from 0 is equal to the starting number of Gaussians, thus implying that all Gaussian components are useful for the final fit). This could imply that the possible ideal number of subpopulations in a distribution is higher than the found value, and the procedure could not be as efficient as it was shown to be in our results.

## Figures and Tables

**Table 1:** Main pipeline tunable parameters, together with their suggested values/ranges according to our experiments.

Algorithm	Parameter Name	Suggested Value ^1^	Suggested Range ^2^
BGM	w.c._prior_type	*dirichlet_process*	-
BGM	covariance_type	*full*	-
BGM	n_components	-	[10, 30]
BGM	tol	$10^{-2}$	[$10^{-5},10^{-1}$]
Lognormal Shrinkage	contraction factor	5	[2, 10]
UMAP	metric	*euclidean*	-
UMAP	nn	50	[30, 500]
UMAP	min_dist	0.0	[0.0, 0.1]
UMAP	init	*spectral*	-
HDBSCAN	min_samples	max(0.005% of cells, 10)	-
HDBSCAN	cluster_selection_eps	∼ 0.1	[0.0, 0.3]
HDBSCAN	cluster_selection_met	*eom / leaf*	-

^1^ A value is reported only if it does not vary over different datasets; if that is not the case, a brief explanation of how to tune it is reported in the result section. ^2^ A range is reported only if different values could be suggested for different datasets.

**Table 2:** Clustering table summarizing performances for BRAQUE (BR) and PhenoGraph (PH) on the 8 different datasets. Where a comparison can clearly be better or worse, the bold value indicates the best algorithm.

Dataset	Whole Lymph Node		L1		L2		L3		T1		T2		T3		CODEX ^2^		Normalized
Algorithm	BR	PH	BR	PH	BR	PH	BR	PH	BR	PH	BR	PH	BR	PH	BR	PH	Average Difference (%) ^3^
**Infrequent populations** **(# out of 15)**	**9**	6	1	1	1	**2**	5	4	4	4	**4**	1	**5**	3	2	2	+5.8
**Common populations (# out of 31)**	**27**	17	**19**	14	**23**	16	**17**	13	**24**	16	**22**	20	**20**	15	**14**	12	+17.3
**% of unclear clusters cells**	**0.2**	7.0	**2.8**	14.2	**0.3**	16.5	**1.2**	36.6	**1.9**	3.8	**2.5**	15.1	**0.0**	1.9	0.9	**0.0**	−10.7
**% of T cells not separated**	**6**	25	1	**0**	0	0	15	**0**	**20**	15	**0**	58	**28**	62	3	**0**	−11.3
**number of clusters**	243	48	89	32	73	29	59	27	106	33	78	35	73	33	89	22	X

^2^ This publicly available dataset had only usable 20 markers. ^3^ This measure indicates the average of (BRAQUE metric—PhenoGraph metric) divided by the maximum possible for that specific metric, may it be 15 for Infrequent populations, 31 for Common populations, or 1 for percentages.

## Data Availability

CODEX Publicly available dataset analyzed in this study can be found here: https://portal.hubmapconsortium.org/browse/dataset/c95d9373d698faf60a66ffdc27499fe1. The datasets whole lymph node, L1, L2, L3, T1, T2, and T3 presented in this study are available at: http://dx.doi.org/10.17632/j8xbwb93x9.1.

## Abbreviations

The following abbreviations are used in this manuscript:

BICCN	BRAIN Initiative Cell Census Network
BGM	Bayesian Gaussian Mixture
BRAQUE	Bayesian Reduction for Amplified Quantization in UMAP Embedding
CyBorgh	(not an abbreviation, onomatopoeic similarity with Simone Borghesi)
DBSCAN	Density-Based Spatial Clustering of Applications with Noise
Dirichlet process	DP
FFPE	Formalin-Fixed Paraffin-Embedded
HCA	Human Cell Atlas
HDBSCAN	Hierarchical Density-Based Spatial Clustering of Applications with Noise
HubMAP	Human Biomolecular Atlas
LNS	Lognormal Shrinkage
MAD	Median Absolute Deviation
MDS	Multidimensional Scaling
MILAN	Multiple Iterative Labeling by Antibody Neodeposition
PCA	Principal Component Analysis
t-SNE	t-distributed Stochastic Neighbour Embedding
TMA	Tissue microarray
UMAP	Uniform Manifold Approximation & Projection
